# Into a Fluoroless Future: an Appraisal of Fluoroscopy-Free Techniques in Clinical Cardiac Electrophysiology

**DOI:** 10.1007/s11886-021-01461-y

**Published:** 2021-03-02

**Authors:** Christopher S. Purtell, Ryan T. Kipp, Lee L. Eckhardt

**Affiliations:** 1 Department of Medicine, Division of Cardiovascular Medicine, Electrophysiology Service, University of Wisconsin-Madison, 1111 Highland Ave, Madison, WI 53792 USA; 2 William S. Middleton Memorial Veterans Hospital, Madison, WI USA; 3 Cellular and Molecular Arrhythmia Research Program, Department of Medicine, Division of Cardiovascular Medicine, University of Wisconsin-Madison, Madison, WI USA

**Keywords:** Electrophysiology, Diagnostic techniques and procedures, Fluoroscopy, Electroanatomic mapping

## Abstract

**Purpose of Review:**

There are risks to both patients and electrophysiology providers from radiation exposure from fluoroscopic imaging, and there is increased interest in fluoroscopic reduction. We review the imaging tools, their applications, and current uses to eliminate fluoroscopy.

**Recent Findings:**

Multiple recent studies provide supporting evidence for the transition to fluoroscopy-free techniques for both ablations and device implantation. The most frequently used alternative imaging approaches include intracardiac echocardiography, cardiac MRI guidance, and 3D electroanatomic mapping systems. Electroanatomic mapping and intracardiac echocardiography originally used to augment fluoroscopy imaging are now replacing the older imaging technique. The data supports that the future of electrophysiology can be fluoroscopy-free or very low fluoroscopy for the vast majority of cases.

**Summary:**

As provider and institution experience grows with these techniques, many EP labs may choose to completely forego the use of fluoroscopy. Trainees will benefit from early experience with these techniques.

## Introduction

Since the mid-1880s when Gaskell reported on the innervation of the heart in a tortoise [1] and Waller demonstrated the electromotive forces induced by a heartbeat [2], the field of cardiac electrophysiology continues to adapt and incorporate technologic advances toward diagnostic and therapeutic techniques [3]. Pioneers in the field of electrophysiology (EP) historically incorporated imaging with fluoroscopy for insertion and manipulation of intracardiac catheters because this was the only imaging modality available. Thousands of trainees were subsequently trained in this modality. However, with the invention and continued evolution of non-fluoroscopic imaging modalities, the heavy reliance on fluoroscopy has been questioned and in most training programs minimized. The current review discusses the general risks of fluoroscopy in the modern electrophysiology lab, examines alternative approaches to fluoroscopy, identifies patient subpopulations that may benefit significantly from these alternatives, and briefly considers the costs associated with adopting this technology as well as the possibilities for its elimination in the EP lab.

## Methods

The literature and research cited herein were obtained from publicly available electronic sources, including PubMed. The search terms included the following: cardiac electrophysiology, diagnostic imaging, cardiac imaging techniques, fluoroscopy, ablation techniques, electroanatomic mapping, and catheter ablation. Additional search criteria included full-text articles, English language, human subjects research, and new articles published within the last 5 years per the editor’s request. Several older studies were included for historical context. Every effort was made to focus our search on cardiac electrophysiology; however, several relevant articles from interventional cardiology and radiology were also included. Methodical cross-checking of available studies was employed, but we cannot rule out that non-public domain research has been performed that we could not include. Emphasis on studies that were primarily focused on fluoroscopy-free techniques was made. This is a topic overview and not intended to encompass all literature related to imaging for cardiac electrophysiology.

## Risks of Fluoroscopy

Despite application of the As Low As Reasonably Achievable (ALARA) principle, there continue to be risks of ionizing radiation exposure to acknowledge when utilizing fluoroscopic imaging [4]. The various effects of this exposure are both stochastic and deterministic with impact on the operator, support staff, and patient. The end result of this exposure can include various forms of malignancy, radiation skin damage, cataracts, and genetic defects [5].

The excess cancer risk attributable to routine ionizing radiation exposure has been estimated to be 1 in 100 among interventional cardiologists [6]. This risk from radiation exposure was demonstrated in a case series investigating head and neck tumors among 23 interventional cardiologists, 2 electrophysiologists, and 6 interventional radiologists. The authors found a disproportionate number of left-sided brain tumors (85%) suggesting these were related to occupational radiation exposure [7]. The risk of cancer may be even higher for female interventional cardiologists compared with male colleagues [8].

Given the risk of radiation exposure from fluoroscopy, application of appropriate safety equipment and shielding can mitigate overall radiation dose. This can include wearable lead aprons and shielding, protective eyewear, appropriate collimation, auto-exposure settings, and other technical improvements [9]. In a single-center cohort study, systematic incorporation of guideline-based interventions reduced radiation exposure by 85% [10]. A similar single-center study comparing a standard low-dose fluoroscopy protocol to a “lowest-dose” protocol in 100 complex left atrial and 40 standard EP procedures showed a 77% reduction in total radiation dose without a significant difference in procedure times or complications between the two groups [11].

Beyond the risk of malignancy, there are several orthopedic concerns associated with routine fluoroscopy imaging due to the need for protective lead shielding. In a survey of members of the Society for Cardiovascular Angiography and Interventions (SCAI), over 40% of survey responders reported significant spine problems, and 28% of responders identified lower-extremity orthopedic problems. The rate of spine injuries appeared related to the number of years spent in practice with 60% of individuals with over 21 years in practice reporting spine problems despite this group only accounting for 20% of responders [12]. Moving to zero fluoroscopy techniques that do not require the use of lead reduces the risk of orthopedic injury throughout the provider’s career.

## Alternatives to Fluoroscopy

**Fig. 1:**
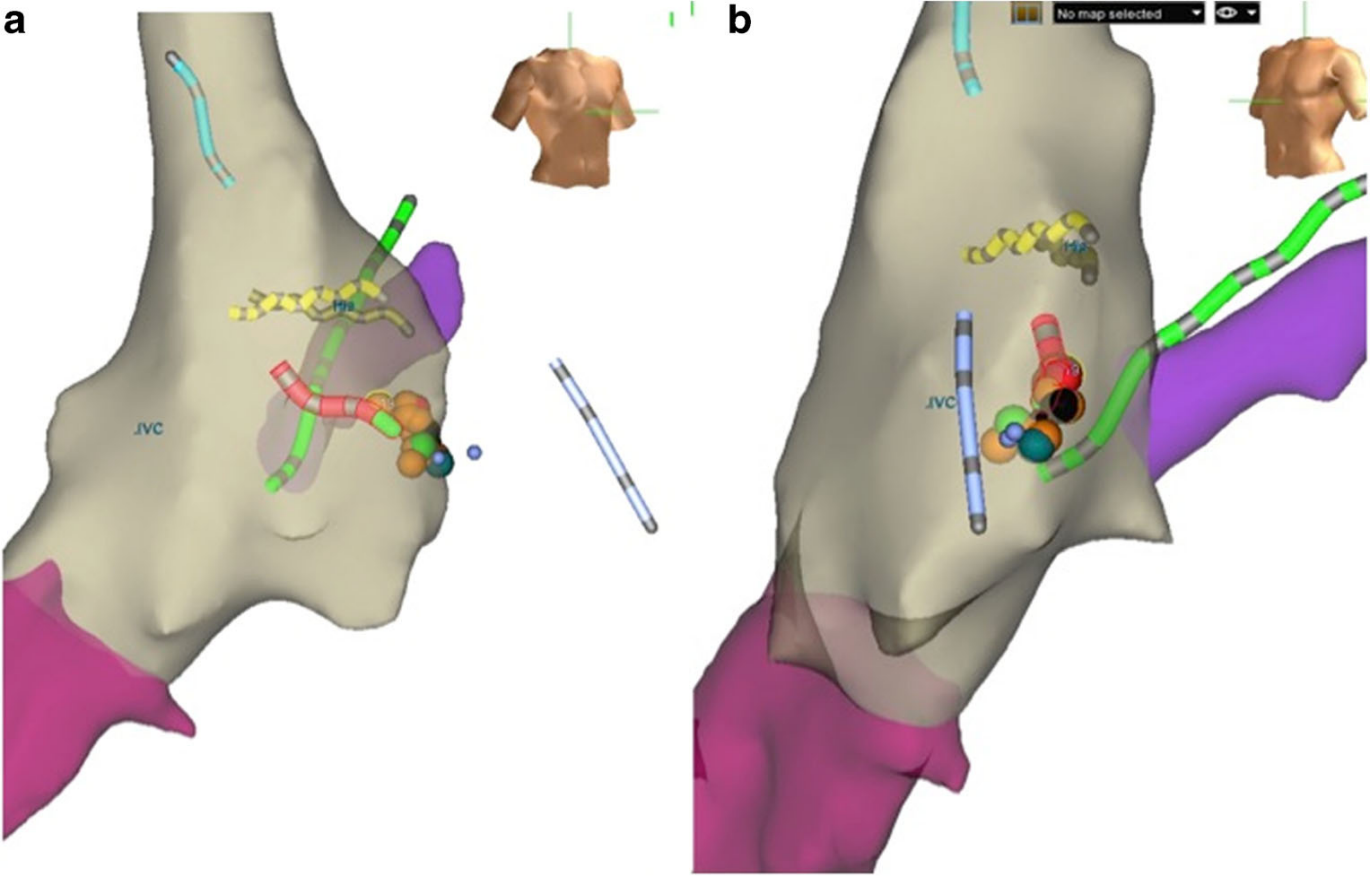
Representative 3D EAM image from an FF ablation for AV nodal reentry tachycardia. The right anterior oblique (**a**) and left anterior oblique (**b**) show right atrial (tan), coronary sinus (purple), and inferior vena cava (fuchsia) reconstruction and respective diagnostic catheters (right atrial in light blue, right ventricle in periwinkle, coronary sinus in green, and His bundle in yellow). The cryoablation catheter is shown in red, with ablation lesions marked by circles (from: Kipp RT, et al. *J Innov Cardiac Rhythm Manage.* 2018;9(9):3305–3311, by permission of MediaSphere Medical, LLC) [24•]

Various alternatives to fluoroscopy have been developed that allow for either minimal or zero-radiation exposure during both simple and complex electrophysiology procedures. These include intracardiac echocardiography, cardiac MRI guidance, and 3D electroanatomic mapping systems (Fig. 1). Intracardiac echocardiography (ICE) is increasingly used to compliment or as an alternative to fluoroscopy for various interventional procedures both within electrophysiology and other structural procedures [13].

### Intracardiac Echocardiography

In the EP lab, ICE offers several advantages over transthoracic and transesophageal echo [14, 15]. Due to the intra-cardiac position of the probe, there are much shorter image distances which allows for higher resolution and image quality. It can also be performed without sedation, unlike the bulky transesophageal echo probe. These devices range from 8F to 10F sizes and have multiple imaging capabilities, including M-mode, 2D, 3D, and color Doppler. These catheters are approved for both venous and intra-arterial use.

**Fig. 2:**
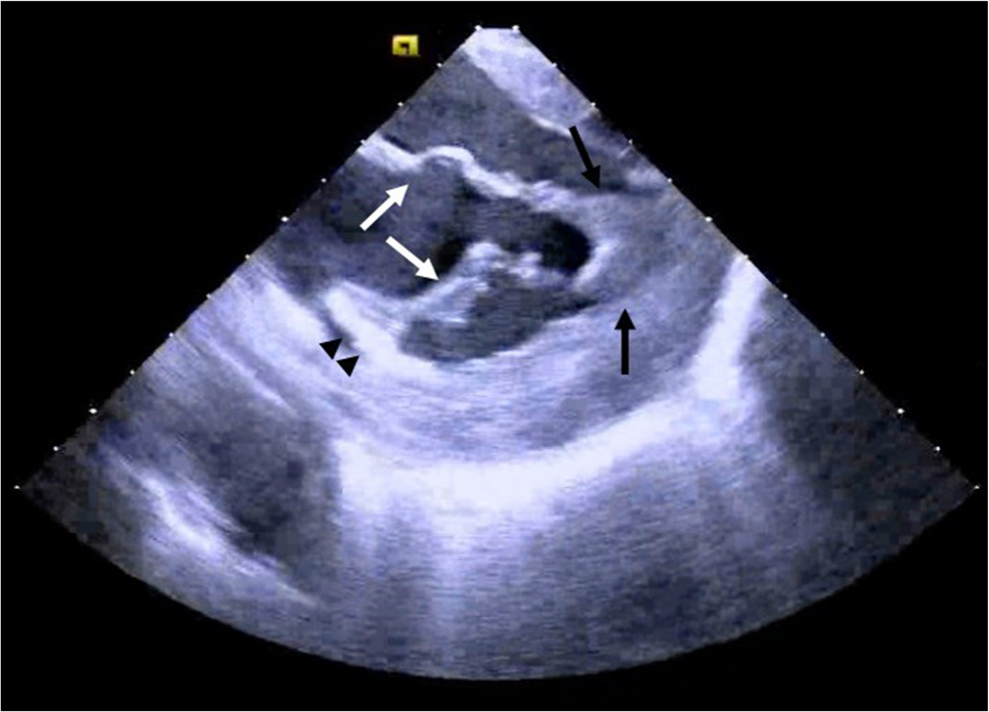
Representative intracardiac echocardiography (ICE) image from ablation of ventricular tachycardia. The black arrows indicate the anterolateral papillary muscle. The white arrows indicate the mitral valve. The black arrowheads indicate the ablation catheter (image courtesy of Ryan Kipp, MD)

ICE is commonly used for procedures requiring transseptal puncture. Unlike fluoroscopy, which relies on determining catheter position against the cardiac silhouette, ICE allows for more direct visual guidance and assessment of difficult septal anatomies [16]. Because an ICE catheter can be manipulated to the region of interest, this imaging technique can also demonstrate more precise tissue contact of catheters, both improving success of ablations and safety (Fig. 2). Additionally, pericardial imaging to identify pericardial adhesions is feasible with this technology [17].

Patient selection for ICE-guided interventions must be made with the understanding that the ICE catheters are large (require 8F to 11F sheathes). This may increase the likelihood of access site complications and vascular injury or may become a procedural hurdle with smaller patients. Technically, the limits of the small imaging field must also be considered, as it limits the ability to see the full interventional field.

### Real-Time MRI

MRI guidance has been incorporated into invasive catheter-based procedures in a number of ways. The most frequently utilized is pre-procedure image acquisition merged with 3D electroanatomic mapping for faster anatomic reconstruction [18]. Real-time MR is an emerging technology that combines visualization of cardiac arrhythmia substrate with real-time visualization and tracking of catheters both during diagnostic and ablation procedures [19]. This technique employs the use of MR conditional catheters that function similarly to conventional catheters but are designed for the MR scanner. Imaging is acquired using a three-dimensional dataset with a navigator and ECG-gated 3D whole-heart balanced steady-state free precession sequence. A unique feature is that active tracking is independent from heart rhythm since the system derives catheter coordinates directly in the coordinate system of the MR scanner independent of the surrounding structures.

### 3D Electroanatomic Mapping

Most all electrophysiology labs have incorporated 3D electroanatomic mapping (EAM) for arrhythmia and anatomic mapping, improved catheter visualization, and improved capability to uncover arrhythmia mechanisms. Presently, there are two main widely utilized EAM vendors that meet the four basic requirements for effective EAM [20] including CARTO (Biosense Webster, Irvine, CA) and EnSite (Abbott Labs, Abbott Park, IL). Other EAM mapping systems such as Rhythmia (Boston Scientific, Cambridge, MA) are in development and becoming more frequently encountered. CARTO and Ensite utilize unique technologies to visualize catheters in 3-dimensions and create the electroanatomic map.

CARTO incorporates three low-level magnetic field–emitting coils. Each of the coils emits a unique frequency which is registered by the tip of a chip-enabled mapping catheter allowing for triangulation of the catheter tip in 3D space. Utilizing the catheter tip position of either single or multipolar catheters, Fast Anatomical Mapping (FAM) can be performed to create the anatomic shell of the chamber of interest. ICE-acquired anatomic images from navigation chip–enabled ICE catheters can also be incorporated into the map to facilitate catheter manipulation and improve understanding of the anatomy. At each site on the FAM shell, a local unipolar and bipolar EGM can be recorded. Utilizing the local voltage, areas of scar and heterogeneous tissue can be identified on a voltage map. Advances in mapping technology including multi-electrode mapping and “Confidense” mapping have facilitated rapid acquisition of points and “Coherent” mapping has enabled improved visualization of this information to identify the best fit activation pattern.

Ensite utilizes impedance technology to localize any catheter within the body. The most recent version of Ensite (Precision) not only uses impedance but also incorporates magnetic field localization technology for improved accuracy. The impedance mapping relies on the application of low-level separable currents that are applied across the patient’s chest. These currents are injected from orthogonal electrode pairs and allows for co-localization of the catheter tip based on the resulting potential difference in the recording tip with respect to a reference electrode. Unlike CARTO, with Ensite the chamber geometry can be determined with any catheter with an electrode, and any catheter can be utilized for mapping and recording of intracardiac signals for the voltage and activation map [20, 21], which is timed against a fiducial point on reference catheter (similar to CARTO). Advanced activation non-contact mapping is available in some systems and utilizes a multi-electrode mesh that is mounted on a balloon and positioned in the cardiac chamber of interest. Without actually contacting the endocardial surface, myocardial potentials can be reconstructed by recording the cavity potentials allowing for indirect reconstruction of the endocardial potentials and activation patterns [20–22].

While these tools facilitate visualization of anatomy and critical areas of arrhythmia generation, it is essential for the electrophysiologist to determine whether the displayed map is plausible or may be introducing unforeseen errors [20, 21, 23]. All of these EAM technologies are independent from fluoroscopy and given that trainees are trained in these techniques, are often the primary mechanism for fluoroscopy reduction in the EP lab.

## Outcomes

With advances in the electroanatomic mapping systems and ICE, the feasibility of fluoroscopy-free ablation has become a reality. The initial procedures performed fluoroscopy-free were right-sided ablations for SVT. Single-center retrospective studies showed that fluoroscopy could be safely withheld during 72–95% of SVT procedures without an increase in procedure duration or complication rate [24•, 25, 26]. These results were confirmed in the multi-center, randomized NO-PARTY study where patients referred for EP Study and ablation were randomized to fluoroscopy guided or minimal fluoroscopy guided ablation with EnSite. There was no difference in the rate of acute or long-term success, nor complications, between the two groups of patients [27].

As understanding of and comfort level with fluoroscopy free ablation improved, fluoroscopy-free procedures were expanded to include more complex, left-sided arrhythmias. Utilizing Ensite or CARTO with either ICE or TEE guidance, multiple single-center, observational studies showed the feasibility of performing fluoroscopy-free pulmonary vein isolation [28–31]. In a randomized study utilizing CARTO and ICE in eighty patients with paroxysmal atrial fibrillation, there was no difference in procedure complications, procedure duration, or acute or 1 year outcomes between patients randomized to ablation with or without fluoroscopy [32].

Recently, the “Ice and ICE” study looked at the safety and efficacy of conventional fluoroscopy-guided cryoablation with ICE-guided cryoablation of AV-nodal re-entrant tachycardia (AVNRT) [33•]. In this small retrospective comparative study, all patients (22 in ICE arm and 25 in fluoroscopy arm) had successful modification of the slow pathway with similar overall procedure times without major complication events reported. However, in that study the ICE-guided group had a longer time for catheter placement, and shorter cryo-application time.

While there have been no randomized trials looking at fluoroscopy-free VT ablation, several case series have been published. In one series, nineteen patients with right- or left-sided idiopathic ventricular tachycardia were ablated using CARTO and ICE without fluoroscopy with a 100% acute success rate (89% success rate at 18 months) and no acute complications [34].

Beyond EP studies and ablation, 3D EAM has been used to reduce radiation exposure during device implantation. Utilizing Ensite for lead visualization, pacemakers and ICDs have been successfully implanted without using fluoroscopy [35–37]. In a larger study, 61 consecutive patients were enrolled to receive a CRT-D either with fluoroscopy (35) or with Ensite mapping alone (26). Remarkably, 92% of the 26 patients underwent successful implantation without fluoroscopy and there were no procedural complications or catheter dislodgement [38].

## Unique Populations

Pediatric electrophysiology has been at the forefront of zero-fluoroscopic techniques over the past decade [39–41]. Given the young age of these patients and their relatively long life expectancy compared with adults, providers must carefully weigh the effects of ionizing radiation exposure. With the curative nature of catheter ablation for a variety of childhood tachyarrhythmias, fluoroscopy-free ablation is an appealing treatment advancement for individuals with and without structural abnormalities [42–44].

While there are no randomized trials of fluoroscopy-free ablation in pediatric patients, in a case series of 63 pediatric patients referred for SVT ablation using CARTO EAM, 54% of the patients received ablation without fluoroscopy [45]. In another series of 5 pediatric patients referred for ablation of idiopathic VT, 3 were successfully performed without fluoroscopy [46].

Pregnancy poses a unique risk for exposure to ionizing radiation. Some data suggest an increased risk for developing arrhythmias during pregnancy. This is likely mediated by changes in autonomics, hormones, and hemodynamics related to pregnancy [47]. Specifically, SVT is relatively common in this population with an estimated incidence of 13–24 per 1000 pregnancies [48]. If ablation is required for arrhythmia control, strategies that mitigate radiation exposure are essential for both the developing fetus and the mother. There have been case studies published of successful fluoroscopy-free ablation of both supraventricular and ventricular arrhythmias during pregnancy [49, 50].

## Technology Limitations and Barriers to Adoption

The advent of fluoroscopy-free techniques has created some challenges in training environments. Although the goal may be to reduce overall radiation exposure, some data suggest that incorporation of 3D EAM into fellowship training may actually increase overall radiation exposure compared with conventional mapping [51]. This is likely due to repeat fluoroscopy use in order to “verify” catheter position and tissue contact. As these techniques become more widely adopted, even this small amount of radiation exposure will likely abate. It is essential that trainees have sufficient familiarity with fluoroless techniques as they go on to practice independently.

Cost is another barrier to adoption as non-fluoroscopic mapping systems are not available in some adult and pediatric electrophysiology labs [52, 53]. Much of this relates to the anticipated costs associated with the addition of 3D EAM systems to the EP lab. Several studies have attempted to analyze the cost-effectiveness of using EAM.

The first study investigated cost effectiveness in 58 pediatric patients. The authors examined cost-effectiveness of EAM using two different methodologies: the alpha value and value of a statistical life. Based on their analysis, the authors concluded that the use of fluoroscopy-free systems was not cost-effective for most countries unless the children’s correction factor is applied [54].

In a separate study, the authors similarly applied an economic analysis of EAM use for ablation of SVT or atrial fibrillation at a single center using alpha value and value of a statistical life [55]. Although both procedures showed a significant decline in effective radiation dose with EAM, the use of EAM was again not considered cost-effective for SVT for most countries; however, it was considered cost-effective for AF due to the larger magnitude of reduced radiation exposure. While these analyses look at the cost of radiation exposure and malignancy, they do not consider the cost of orthopedic injury as a consequence of protective shielding or the potential costs of radiation exposure to the proceduralists.

Furthermore, the authors of the NO-PARTY study, which compared fluoroscopy guided or minimal fluoroscopy guided EP Study and ablation with EnSite, concluded that the additional cost of incorporating this mapping system was offset by the reduction in cancer afforded by this technique [27].

## Forward-Looking Directions and Conclusions

A plethora of case studies and prospective, randomized controlled trials have shown the effectiveness and safety of implementing a zero-fluoroscopy approach in the electrophysiology lab. The most vulnerable populations, including children and pregnant patients, are probably best served by using a zero-fluoroscopy ablation approach. As centers and providers become more familiar with this technology and these techniques, it is likely that we will see expanded adoption more broadly.

While historically fluoroscopy has been used for imaging guidance, the abundance of non-fluoroscopic imaging techniques provides better anatomic and electrical detail without the risk, cost, and hassle of fluoroscopy. This begs the question of whether or not incorporation of costly fluoroscopic imaging needs to be pursued in EP labs of the future. With more image sophistication as well as early adoption in training or practice, EP providers may feel less and less need to verify position or anatomy by fluoroscopy. From the patient’s perspective, this may mean the invention of EP procedure tables that are more comfortable than a fluoroscopy table.

Education of the next generation of trainees is pivotal in transformational technology adoption. Since 2013 our EP lab has promoted zero to near-zero fluoroscopy early on in the training of new fellows most of whom continued this pattern into their practice following graduation. Comprehensive instruction in multi-imaging modalities for training fellows optimally helps shift the field of EP toward a fluoroless future.
